# The preference for belief, issue polarization, and echo chambers

**DOI:** 10.1007/s11229-022-03880-y

**Published:** 2022-09-29

**Authors:** Bert Baumgaertner, Florian Justwan

**Affiliations:** 1 Department of Politics and Philosophy, University of Idaho, Moscow, USA

**Keywords:** Preference for belief, Preference for certainty, Echo chambers, Epistemic bubbles, Homophily, Issue polarization, Mechanisms of exclusion, Motivated reasoning

## Abstract

Some common explanations of issue polarization and echo chambers rely on social or cognitive mechanisms of exclusion. Accordingly, suggested interventions like “be more open-minded” target these mechanisms: avoid epistemic bubbles and don’t discount contrary information. Contrary to such explanations, we show how a much weaker mechanism—the preference for belief—can produce issue polarization in epistemic communities with little to no mechanisms of exclusion. We present a network model (with an empirically-validated structure) that demonstrates how a dynamic interaction between the preference for belief and common structures of epistemic communities can turn very small unequal distributions of initial beliefs into full-blown polarization. This points to a different class of explanations, one that emphasizes the importance of the initial spread of information. We also show how our model complements extant explanations by including a version of biased assimilation and motivated reasoning—cognitive mechanisms of exclusion. We find that mechanisms of exclusion can exacerbate issue polarization, but may not be the ultimate root of it. Hence, the recommended interventions suggested by extant literature is expected to be limited and the problem of issue polarization to be even more intractable.

## 1 Introduction

Issue polarization may be even more intractable than common explanations of it suggest. If polarization emerges due to motivated reasoning, such as biased assimimilation or the backfire effect, one intervention might be at the level of individuals, e.g., educate individuals to better update their beliefs. We know, however, that even if individuals follow some rational belief updating guidelines, such as some variety of Bayesianism, that is no guarantee that consensus will form: individuals themselves may be rational, but the social means by which beliefs are shared and aggregated can prevent consensus. Some of these social forces may be exclusionary (e.g. Nguyen, 2020), others may be more subtle by co-opting the same mechanisms that make the spread of true beliefs possible (e.g. O’Connor et al., 2019). To boot, whatever the relevant social forces are, social media can further facilitate and exacerbate their contribution in generating issue polarization. Perhaps, then, a better intervention is directed towards the relevant social forces, or perhaps both, resources permitting. Alas, the problem, to the extent that it is one, may have other more nuanced causes.

From a very high vantage, here is what is to come. We will illustrate how well-formed epistemic communities composed of reasonable cognitive processes will still be susceptible to issue polarization. The epistemic communities are well-formed in that the connections between agents are not exclusionary: equal testimonial status is given across a diversity of values. The reasonableness of the cognitive process lies in its generality: it has widespread empirical support and seems normatively plausible, capturing a shared tenet of numerous schools of thought concerning belief revision. And yet, issue polarization *can* still emerge because of a dynamic interaction between a banal ordering effect at the individual level and the distribution of testimony across epistemic networks; polarization *will* emerge unless information enters the network under just the right symmetrical conditions (which are empirically implausible). If correct, interventions on polarization that depend on identifying some epistemic malpractice will fail.

There are three main parts to our analysis. First is the domain of propositions over which issue polarization occurs, the criteria being that the propositions are: (i) epistemically truth evaluable, but not too easily fact-checked, and (ii) ideologically relevant. Section 2.1 discusses this in more detail.

The second part of our analysis concerns the cognitive process, which we call *the preference for belief*. Leaving details to Section 2.2, the rough idea is that in the absence of defeaters, individuals tend to (and are warranted/justified to) believe what someone in their epistemic community invites them to believe. Of course, motivated reasoning can shape this belief formation process, and we additionally consider this possibility.

The third part of our analysis is on epistemic communities. Individuals have some tendency towards forming epistemic connections with individuals of similar mindedness. ‘Some tendency’ is crucial here, as it is not meant as a malpractice, such as the exclusion of other viewpoints. We imagine that individuals are not only highly tolerant of others with dissenting ideologies, but will also take testimony from them on par with testimony from like-minded peers. All we have in mind by ‘some tendency’ is that on aggregate individuals will have a slightly higher ratio of like-minded connections to different-minded connections. Section 2.3 lays this out in detail.

We bring these three parts of the analysis together in a formal network model. We take care to ensure that the network structure is empirically-validated. We then simulate the model, looking for patterns of how the preference for belief affects the flow of information on the network. What we find is that our epistemic communities tend to preserve the distribution of the initial testimonies when information first enters the network, which, on the one hand seems perfectly reasonable, but on the other means that polarization can be a by-product of epistemic processes that are otherwise free of malpractice. This is crudely put here and Section 3 carefully walks through a detailed examination to make this clear and appropriately qualified (with further clarifications and qualifications in Sections 4 and 5).

With a better understanding of the kinds of epistemic communities featured in our analysis, we consider some broader implications in our last section. Numerous mechanisms have been identified that plausibly contribute to polarization and the related phenomenon of echo chambers. Many of these extant analyses make assumptions similar to the ones we consider here, but then additionally add some mechanism thought to be at fault (motivated reasoning, exclusion, biased assimilation, etc). Such accounts (implicitly) suggest an intervention related to the explanation. But if we are right, then such interventions are not expected to work, at least not to the extent that our model represents what actual epistemic communities could aspire to. While we briefly consider interventions that would be consistent with our analysis, we surmise that issue polarization is simply too intractable to solve.

## 2 Analysis of parts and model assumptions

We divide our analysis into three parts, providing motivation and clarification for how we are thinking about them. Where appropriate we also explain how they are formally implemented.

### 2.1 Beliefs of interest

Consider a proposition S that is difficult to verify, but also has the feature that its truth value is either congruent or incongruent with an ideological worldview.

With respect to the first point, some propositions are more easily fact-checked than others, and resources exist that make such fact-checking more easily accessible for the non-expert (snopes.com is one example). Other propositions are more difficult to verify by the non-expert, but can be fact-checked by those with the relevant expertise. Still other propositions are simply too difficult to support with bare evidence and require appeals to counterfactual reasoning. For example, compare statements S1 and S2:

S1 Government X engaged in an “influence campaign” involving the use of fake Facebook accounts and Twitter bots in an attempt to affect the outcomes of the Presidential election in Government Y.

S2 Government X’s “influence campaign” decided the election in Government Y.

By substituting the names of actual governments for X and Y, we can then fact-check S1 by verifying that, e.g., i) there are Facebook accounts and Twitter bots that are owned by Government X, ii) that these accounts put forward content that favored one candidate in the Government Y election over another candidate, and iii) that Government X has a motive to engage in such behavior. But now compare that to what it would take to fact-check S2. While S1 is a claim about whether there was or wasn’t the relevant influence campaign, S2 is a claim that such a campaign played a decisive role in the success of one of the candidates, i.e. that the influence campaign is the reason that explains why the winning candidate won. Trying to fact-check S2 requires something like verifying the counterfactual claim that if there hadn’t been an influence campaign by Government X, then the election would not have been decided for them. Whatever the method for fact-checking, the point is that verifying S2 is inordinately more difficult than S1, if it is even feasible at all.

This brings us to the second feature. Despite the difficulty of fact checking statements like S2, they can be congruent or incongruent with ideologies. Consider, for example, how presidential candidates often represent ideological world views. For simplicity and illustration, suppose that Candidate Purple represents the Purple ideology (the “left”), and Candidate Orange reflects Orange ideology (the “right”). Moreover, suppose that if a candidate wins an election, supporters of that candidate are likely to say that the election was fair, while supporters of the losing candidate are less likely to say it was fair. So if Candidate Orange wins in Government Y’s election, those of the Orange ideology are less likely to agree with S2 than those of the Purple ideology. This feature, that ideological congruence impacts various evaluations of democratic performances, has strong empirical support (see Singh et al., 2011).^1^

The connection to ideology means there are least two kinds of preferences that can impact people’s beliefs about propositions like S. One is in terms of motivated reasoning. This might mean that different pieces of information are discounted to be made appropriately congenial to the ideology. It can also mean the selective use of intellectual capacities when presented with information. Motivated numeracy, the selective use of quantitative-reasoning to conform interpretations of data to (political) outlooks, is an excellent example of this (see Kahan et al., 2017). The second kind of preference in which ideology can present itself is in the selection of one’s epistemic community. Political homophily, for example, is a preference for forming connections with politically like-minded individuals and can affect how beliefs spread. We discuss these two kinds of preferences in turn and the way we incorporate them in our model.

### 2.2 The preference for belief and motivated reasoning

As a first pass, the *preference for belief* can be stated as follows: in the absence of defeaters, individuals tend to come to believe what someone in their epistemic community invites them to believe. It will be helpful to look at similar accounts of belief formation and updating. The goal is not to give a fully fleshed out account of the preference for belief. Rather, the point is to identify that what is shared with all of the examples is that they have the same dynamic structure that is susceptible to a banal ordering effect.

Perhaps the most similar example to the preference for belief is Tyler Burge’s discussion of the Acceptance Principle: “A person is entitled to accept as true something that is presented as true and that is intelligible to him, unless there are stronger reasons not to do so.”(Burge, 1993). One key difference, however, is that we take the preference for belief to be descriptive rather than normative—though not much hangs on this. Moreover, the Acceptance Principle is not explicitly inter-personal (textbooks might “present”, or the person might think up the proposition themself).

Another example comes from Gilbert Harman regarding what he calls the Principle of Positive Undermining: “One should stop believing P whenever one positively believes one’s reasons for believing P are no good.” (Harman, 1986, p. 39) While this example is about how to approach existing beliefs after we may have forgotten about how we got them, as opposed to what beliefs to form in the first place, the point is that once we have a belief, the burden is on providing a reason to stop believing as opposed to continuing.

Similarly, in contrasting radical probabilism to foundationalist epistemologies, Simon Huttegger notes that they share features for how they view belief revision: “According to general foundations theories, at least some of an agent’s beliefs at a time are taken to be justified by default or until proven incorrect. *Belief revision changes these initial beliefs only in the face of sufficiently strong evidence.* The inductive assumptions of an agent can be taken as beliefs of this sort whenever they represent her fully considered opinions. Being justified by default means that as long as the evidence does not speak against them, probabilities are revised by new observations so as to be consistent with those inductive assumptions.” (Huttegger, 2017, p. 105, emphasis ours)

These examples so far are primarily individualistic. Something like an explicit inter-personal version of the preference for belief is discussed in social epistemology, particularly with respect to testimony. For example, in *Knowledge in a Social World* Alvin Goldman considers different kinds of practices for how and what people report to one another regarding their beliefs, and relatedly, different acceptance practices for how individuals go about adopting beliefs given the reports (Goldman, 1999). One naive acceptance practice is called BLIND TRUST, which is to believe every report you hear.^2^ While BLIND TRUST might work well in some contexts, e.g., ones in which only truths are generated in the reporting practices of a community, it is certain to fail in other contexts, e.g. ones where only falsehoods are generated. To optimize the spread of beliefs that are true, we would want to endorse particular *pairs* of acceptance and reporting practices, e.g. BLIND TRUST with ONLY-SAY-WHAT-YOU-BELIEVE-TRUE. Alas, a practical point of view shows this to be an excessive demand, for there are many diverse reporting contexts and it would be intractable to determine which specific acceptance practice is optimal for each reporting context. Goldman thus suggests that we endorse practices that produce veritistic improvements on average. He argues that a supplemented version of Bayesian inference that applies to testimony best satisfies this more reasonable demand. One potential criticism, however, is that the move towards Bayesian inference might itself be too high a demand. That aside, Goldman’s work is again a normative enterprise, whereas the preference for belief is meant to be descriptive.

The preference for belief is not just a philosopher’s idea. It is consistent with the mimetic hypothesis of preferences.^3^ The neoclassical economic view of preferences is that individuals are sovereign with respect to want what they want, as if individuals know with a high degree of certainty exactly what they want under all circumstances. By contrast, the mimetic hypothesis says that in many instances we look towards others as a model to imitate. The mimetic hypothesis is able to explain how individuals are able to influence each other, how individual preferences can often reflect their social environment, and in an economic context, how an increase in the price of a commodity does not necessarily drive down demand (as predicted by the neoclassical view of preferences) but can actually increase demand. In brief, in many situations we either do not have our own preferences, or if we do, we do not know what they are. We consequently look towards others to develop or learn our own preferences. In the context of the preference for belief, agents would look towards others to form many of their beliefs, particularly in the absence of being able to make their own observations. That is the context that propositions like S2 find themselves in: they are difficult to independently verify and agents will look towards others as models for what to believe.

Finally, from a descriptive perspective, people *do* seem to adopt what they first hear if they do not have defeaters. For example, at the intersection of psychology and linguistics there is a long standing tradition that argues that people tend to believe the messages they hear.^4^ This is often supported by evolutionary arguments: in environments where information tends to be accurate a propensity to believe is efficient (see Reber and Unkelbach (2010) and Kissine and Klein (2013)). Or consider, for example, the phenomenon of belief perseverance, in which subjects form beliefs from (false) reports and then continue to believe many of those things *even after subjects acknowledge the discrediting of the evidence* on which they initially formed their beliefs (Ross & Anderson, 1982). And it seems that the political context is no exception: people are prone to believing anything that they read (Pantazi et al., 2021).

The preference for belief, and the other examples just discussed, have a dynamic structure that is susceptible to an ordering effect as follows: (i) an individual is invited to adopt a preference, belief, value, or mental state, (ii) the individual does not have a mental state that would be incongruent with what they are invited to adopt (call this the “why-not” element), and (iii) after having adopted whatever was on offer, alternative offerings that are on par are not accepted. For example, suppose F and G are incongruent beliefs and an agent does not have any reasons against either. Suppose our agent is invited to believe F by someone in her epistemic community. By the why-not element, she adopts F. Now if she were presented with G, she would not accept it since F is incongruent with it—the why-not element has been removed. The key feature of this dynamic is that had G been presented first, our agent would have adopted it instead of F and the situation would be reversed.

To be clear, this is not to say that our agent would never accept the alternative. Absent additional considerations the adoption of F means that G will not be accepted, but of course additional evidence or reasons might be provided to support G, and thereby supplant F.^5^ Since we are limiting ourselves to the kinds of propositions discussed in Section 2.1 which primarily depend on testimony, our agents cannot supplant F by independently gathering evidence for G (or vice versa). However, in Section 4 we consider the possibility that F (and likewise G) could be supplanted by appeal to social considerations.

In addition to the preference for belief, stronger assumptions can be made about how agents process information. There is an extensive literature on *biased assimilation* or *motivated reasoning*. Starting from the work of Lord et al. 1979, the basic idea is that agents treat new reasons as a function of what they antecedently believe, with varying levels of bias in how agents evaluate and selectively adopt evidence. Recent empirical work suggests that under certain conditions ideology is expressly linked to motivated reasoning (Kahan et al., 2017).

In our model we allow for the possibility that the acceptance or rejection of a proposition is also driven by the motivations associated with the relevant ideology. So while our default assumption about the preference for belief is weaker than that of motivated reasoning, we will explore how the addition of motivated reasoning can impact how beliefs about statements like S2 could be spread. More specifically, we add a parameter called *motivated bias*, the probability that an agent declines an invitation to accept a belief that is incongruent with their ideology. We will explore two versions of it: i) uniform motivated bias, where all agents have the same level of bias, and ii) differential motivated bias, where the amount of motivated bias increases as a function of ideological strength.

With this background in place, the belief formation practice of our synthetic community proceeds as follows. Our agents have three belief states: empty-belief, belief-yes, and belief-no. By default at the beginning, all agents have empty-belief, i.e. they have not formed a belief about some proposition like S (this is like the why-not element). A few agents in the community receive “news” about S, and some will form belief-yes and others belief-no. Who comes to believe what will be something we explore in more detail below. From these initial seeding conditions, the process of belief spread consists of cycling through the following two steps:

1. Agents with belief-yes or belief-no invite their empty-belief friends in their epistemic community to accept their belief, *b*.
2. Agents with empty-belief and being invited to believe *b* will either: i) adopt *b* if it is congruent with their ideology, or ii) if *b* is incongruent with their ideology, adopt *b* with probability 1 minus *motivated bias*.

In a “well-mixed” population each agent would have the same chance of interacting with one agent as any other agent. We know for a fact that this is not true in the real world, including social media where many epistemic communities now reside. Rather, communities, online and off, tend to have particular kinds of structure that forms a network of interactions. In the next section we describe in detail the relevant structure(s) of our model.

### 2.3 Network structure and epistemic communities

Rather than speculate, we look towards actual online communities to identify the kinds of structures that epistemic communities typically have. Online communities tend to have two features. First, they have short path lengths between any two nodes. That is, the number of links needed to draw a path from one friend to another is small (think of “seven degrees of separation”—but even shorter for online communities). Random graph networks are paradigmatic examples of having short path length, whereas regular networks that form a ring lattice have longer path lengths. The second feature is clustering, which is when your friends are also friends with each other (sometimes also referred to as transitivity). Small-world networks have both of these features: they have short path lengths and high degrees of clustering. Because we are interested in understanding the preference for belief in epistemic communities in general, we use a stochastic algorithm to generate our small-world networks. We do this to avoid the possibility that one or more idiosyncrasies of a single network have a disproportionate impact on our results.

An extant analysis of Facebook groups (Wohlgemuth & Matache, 2014) allows us to identify some additional details in order to simulate networks. Online communities can vary in size, going as low as 20 (for communities under the search term “Navy”) and reaching up to 1500 (for communities with the search term “religion”). The number of links between “friends” grows as a function of the number of agents (*N*) in the communities, so that the average degree of a network is approximately *N*^0.6^. For example, in a community of 200 agents, agents would have on average about 24 connections, and the community as a whole would have about 2200 connections.^6^ Those connections are distributed so that a small world network is formed, i.e., they have short path lengths and high degrees of clustering.

In addition to these more general features, we also have our network model reflect people’s ideological positions and their general perceptions about their communities. With respect to connections between ideological positions, we draw on Boutyline and Willer (2017), who find that individuals that are ideologically more extreme tend to have higher levels of political homophily in their social networks.

In order to inform our model with respect to people’s perceptions of their communities, we rely on behavioral data extracted from a nationally representative online survey of the U.S. adult population. In particular, we partnered with Qualtrics (a provider of samples for academic research) and we collected 1,520 responses from April 8 to April 27, 2020. The final sample was designed to match the overall demographic breakdown of the United States in terms of age, gender, education, income and Census region. (Prior to launching our survey, we obtained IRB exemption from our institution.)

In order to provide some empirical validation of our model, we first asked our survey-takers to place themselves on a one-dimensional ideological spectrum. Answer options were: (1) very liberal (10.2 percent), (2) liberal (18.5 percent), (3) moderate (37.1 percent), (4) conservative (20.5 percent), and (5) very conservative (13.7 percent). Furthermore, we also asked those respondents who indicated that they had an account on a social media platform (84.4 percent) to reflect on the political diversity of their online network. To this end, we rely on a survey item adapted from Allcott and Gentzkow (2017). More specifically, we instructed individuals to think about their network of online “friends” and other people they follow or interact with on social media. We then asked subjects to estimate what share of friends preferred the same presidential candidate as they did in 2016 (what we call the level of their “bubble”). This “bubble” variable ranges from 0 to 100. Its mean value is 58.9. The standard deviation is 26.0 which suggests a sizeable degree of dispersion. As expected, the data show that ideologues create more congruent social media environments than moderates. In our sample, moderates estimated that about 54.0 percent of their social media friends preferred the same presidential candidate as they did in 2016. For liberals or conservatives (that is, for respondents with ‘weak’ ideologies), the corresponding value is substantially higher (62.4 percent). Finally, for survey-takers with strong political ideologies (subjects who self-identify as “very liberal” or “very conservative”), the self-reported share of like-minded social media friends is 68.5 percent.

Informed by these empirical considerations, we build our network models so that we have an ideology spectrum that goes from 1 to 5, with the characteristics specified in Table 1.

To be clear, we use our empirical data to inform how to build the *structure* of our epistemic communities, i.e. who tends to interact with whom. The interactions themselves are testimonial and governed by the preference for belief. Importantly, the nature of the interaction links between agents are uniform: a link between two moderates means the same as a link between a very left and a very right, etc. That is, our agents do not condition their acceptance of a belief on the source of the testimony. If they condition at all, is it only when there is motivated bias, and that is based on the nature of the information itself and its congruency with their ideology.

## 3 Analysis of polarization and simulation results

We structure the discussion of our results and analysis of issue polarization in two sections that reflect the following ordering of questions of interest:

- Could network structure by itself produce some level of polarization? (Section 3.1)
- Relatedly, how much does the clustering feature of small-world networks, compared to random graphs, impact polarization? (Section 3.1)
- Does motivated bias contribute to polarization? (Section 3.1)
- How sensitive is polarization to initial starting conditions of how beliefs are seeded in the network? (Section 3.2)
- How does(differential) motivated bias contribute to polarization for different starting conditions? (Section 3.2)

To perform simulations, we proceed as follows.^7^ First, for each simulation, the network would be built to have (on average) the features specified in Sections 2.2 and 2.3. Second, four agents would be “seeded” with initial beliefs of belief-yes and belief-no, while all other agents would start with empty-belief (different initializations were extensively explored—see Section 3.2). In our simulations and visualization, we make it so that belief-yes is congruent with purple, and belief-no is congruent with orange. Finally, beliefs would then spread according to the rules specified in Section 2.2.

At the end of a simulation, we look for whether and how much our epistemic community is polarized. There exist different meanings and measures of polarization (Bramson et al., 2017). We are interested in issue polarization, and in particular correlations between the content of a belief and the ideology of the agent that holds the belief. Consequently, what we measure is the frequency of belief-yes conditioned on the ideology of the agents. The degree of polarization in a population depends on the difference in the probabilities of belief-yes given ideologies. For example, if probabilities of belief-yes given each ideology is about 0.5, then there is no issue polarization, since there is no correlation between beliefs and ideology. But if the purple agents have a belief-yes probability of 0.9 and the orange agents have a probability of 0.3, then there is a high degree of polarization. Mutatis mutandis for belief-no, though we omit those results since they are equivalent under symmetrical transformations.^8^ Note that under this kind of measure, echo chambers are intimately tied to polarization: a high frequency of belief-yes counts as an echo chamber when: i) conditioned on one side of the ideological spectrum it is high, and ii) when conditioned on the other side it is low. We leave additional comments about connections to echo chambers to later.

### 3.1 Motivated bias and network structure

Our first set of simulations are meant to answer our questions about the role of network structure in producing polarization and to what extent this is exacerbated by (uniform) motivated bias. To do this we controlled for how the initial beliefs were seeded. In the Arbitrary Start scenario we let the initial seeding beliefs be random, with the only condition that there be at least one of each of the two beliefs. In the Arranged Start scenario we ensured congruence between initially seeded beliefs and ideologies, i.e. the belief-yes beliefs were seeded among purple agents, and the belief-no’s among orange.

**Fig. 1:**
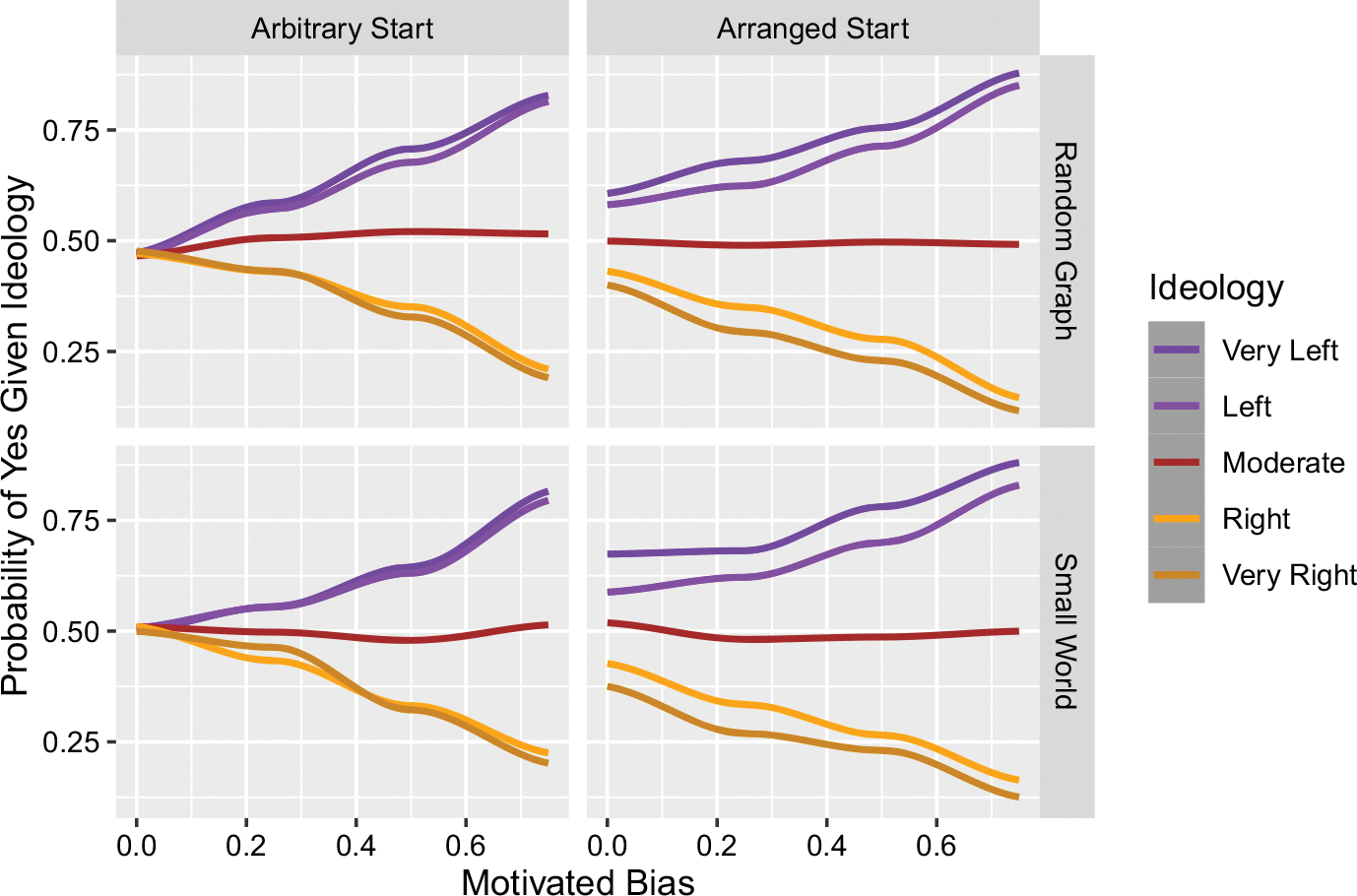
Motivated bias can both produce and amplify existing polarization. Notice, however, that some polarization can happen even when there is no motivated bias, as shown in plots in the right column under Arranged Start. The top row shows simulations done on random graph networks, the bottom row on small world networks. Actual simulated levels of motivated bias were 0, 0.25, 0.5, and 0.75. Each parameter setting was simulated 200 times

Figure 1 demonstrates how motivated bias can both produce polarization and amplify existing polarization. For example, in the Arbitrary Start scenario, it is equally likely that a seeded Left (orange) ideology starts with belief-yes as it is that a Right (purple) ideology does, and when motivated bias is zero those initial beliefs spread randomly in the network ending with an equal chance that agents have belief-yes regardless of their ideology. But as motivated bias increases from zero, we see that it produces increased levels of polarization, i.e. the probability that an agent has a belief-yes given that they have a Left ideology increases, while this decreases for Right ideologies. We see that the polarizing effect of motivated bias is similar for both the random graph network and the small world network.

The fact that motivated bias can lead to polarization is not surprising. What is more interesting is that if we consider the Arranged Start scenario, in which the seed agents start with beliefs that match their ideology, we see that polarization emerges even when motivated bias is zero. We also see that this effect seems to be more pronounced in the small world network than in the random graph.

Our interpretation of this result is as follows. Notice that in our model ideologies do not exclude others simpliciter. While individuals have some preference for connections with like-minded individuals, they have and are just as willing to listen to any one of their friends as another, even if that information is incongruent to their ideology, and even if that friend has an ideology incongruent with theirs. Moreover, when motivated bias is zero, individuals *never* decline an invitation to believe something. So neither of two standard mechanisms of polarization and echo chambers—motivated reasoning and exclusion—are predominantly at work (see Section 5 for further discussion).^9^ And yet, we still find the emergence of polarization.

What explains polarization in our model, even when there are no mechanisms of exclusion, is twofold: first, there is a dynamic interaction between the ordering effect of the preference for belief with an ideologically-distributed structure in an epistemic community, and second, this dynamic interaction magnifies a very small unequal distribution of beliefs that already exists at the start. Importantly, these starts aren’t really “polarization” at all, since just 2% of the population starts with any sort of belief. Even calling it “nano-polarization” could be misleading, but it does convey the initial seedlings that give way to the more full-blown and properly called phenomenon of polarization. The point is that the process is quite subtle. The fact that a purple agent has a few more connections with other purple agents than they do with orange agents, makes it more likely that they will receive an invitation to believe something from a purple agent than an orange one. But that by itself is not enough—if that were all there was, this wouldn’t give us polarization because it doesn’t impact the initial distribution of the seeding beliefs: if you look at the Arbitrary Start scenarios in Fig. 1 when Motivated Bias is 0, the resulting probability of holding belief-yes given ideology is about 50%, the same as how they are seeded on average. What we additionally need is for the initial beliefs, few as they may be, to be distributed unevenly across the ideologies. The dynamic interaction between the belief-spreading process and the network structure then magnify the initial distribution to the community level. This is what we see when looking at the Arranged Start scenarios when Motivated Bias is 0.

This result prompts us to look in more detail how polarization might be sensitive to how beliefs are seeded in a network.

### 3.2 Echo chambers and starting conditions

There are a number of different ways to seed our epistemic communities with beliefs. We do not explore them all exhaustively, for many of them are uninteresting or irrelevant (e.g. if there are only belief-yes to start, then that is all that can spread). To be efficient in our presentation and keep things organized, we adopt the following convention. Upper case letters indicate a token belief-yes, while lower case letters indicate a token belief-no. The letters “p”, “o”, “m” stand for purple (Left), orange (Right), and Moderate, respectively. By combining these designations, we can succinctly represent starting conditions. For example, “ppOO” represents the starting condition where two belief-no were given to the Left (purple) and two belief-yes were given to the Right (orange). “PppO” represents that there is one purple agent with belief-yes, two purple agents with belief-no, and one orange agent with belief-yes. And so on.

We consider two kinds of starting conditions: the first kind has two belief-yes and two belief-no, the second kind has three of belief-yes and one belief-no.^10^

In addition, we weaken an assumption about motivated bias: in the case of the uniform version, a Very Left (deep purple) and a Left (purple) are equally biased, but it seems reasonable to think that motivated bias is a function of the strength of the ideology. Or put different, it seems unreasonable to expect that a Moderate would have as much motivated bias as a Very Left. Thus when we consider how motivated bias impacts the foregoing results, we consider the more plausible case of differential motivated bias. Unless otherwise noted, we made the strong ideologies have a level of 25% motivated bias, the weak ideologies half of that, and the moderates had no bias.

#### 3.2.1 Two yes and two no

**Fig. 2:**
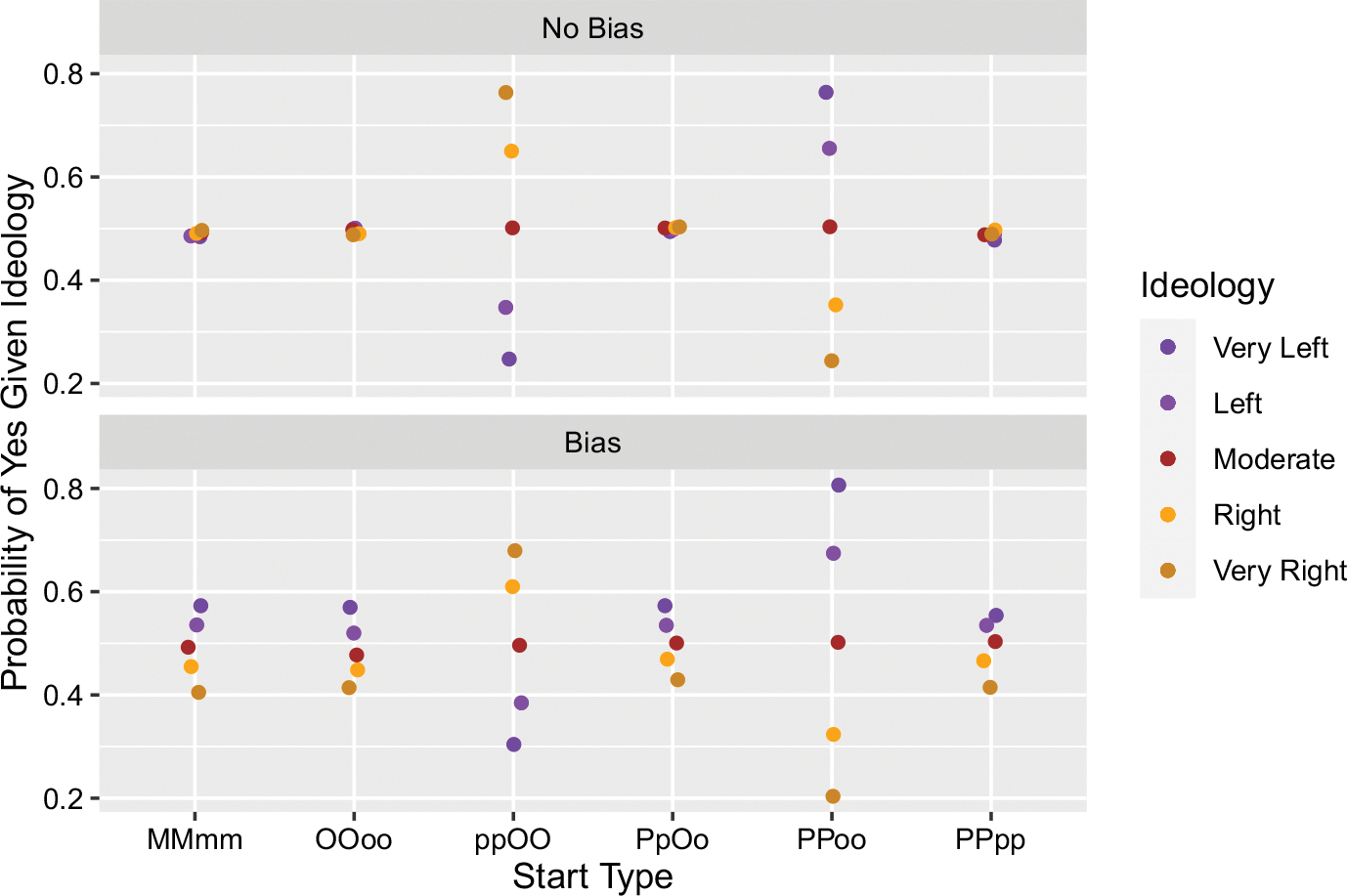
Results from simulations where two “Yes” beliefs and two “No” beliefs were initially distributed in different ways. Upper case letters indicate a token belief-yes, lower case letters a belief-no, and the type of letter indicates ideology type (M = moderate with belief-yes, o = (orange) right ideology with belief-no, P = (purple) left ideology with belief-yes, etc). “Noise” on the x-axis is for illustrative purposes only to avoid dots completely covering one another. Each parameter setting was simulated 1,000 times

Figure 2 shows six different starting conditions in which two belief-yes and two belief-no were seeded in the community. One set of simulations was done without differential motivated bias, the second set with it.

Notice that if the beliefs are seeded equally in a group (MMmm, OOoo, PPpp) or across two groups (PpOo), there is no “echo chamber” and correspondingly no polarization. If, however, we differentially seed the beliefs across groups, as in the cases of ppOO and PPoo, then the resulting probabilities of believing Yes given ideology reflect the initial distribution. In short, the purples tend to spread around what they were initially seeded with, and the oranges theirs. We thus corroborate our interpretation of the results we discussed above: polarization can happen from a dynamic interaction between the preference for belief and ideological structure that magnifies initial conditions.

The introduction of differential motivated bias has three noteworthy effects. First, where there was no polarization in its absence, motivated bias is sufficient for creating it (see MMmm, OOoo, PpOo, and PPpp). Second, where polarization emerged in its absence and the respective “echo chambers” held beliefs congruent with their ideology, as exemplified in the case of PPoo, motivated bias widened polarization. Third, where polarization emerged in its absence, but the beliefs were incongruent with the respective ideologies of the “echo chambers”, motivated bias was not enough to avoid this incongruency. This is shown in the case of ppOO. We will examine this third effect in more detail shortly.

#### 3.2.2 Three yes and one no

**Fig. 3:**
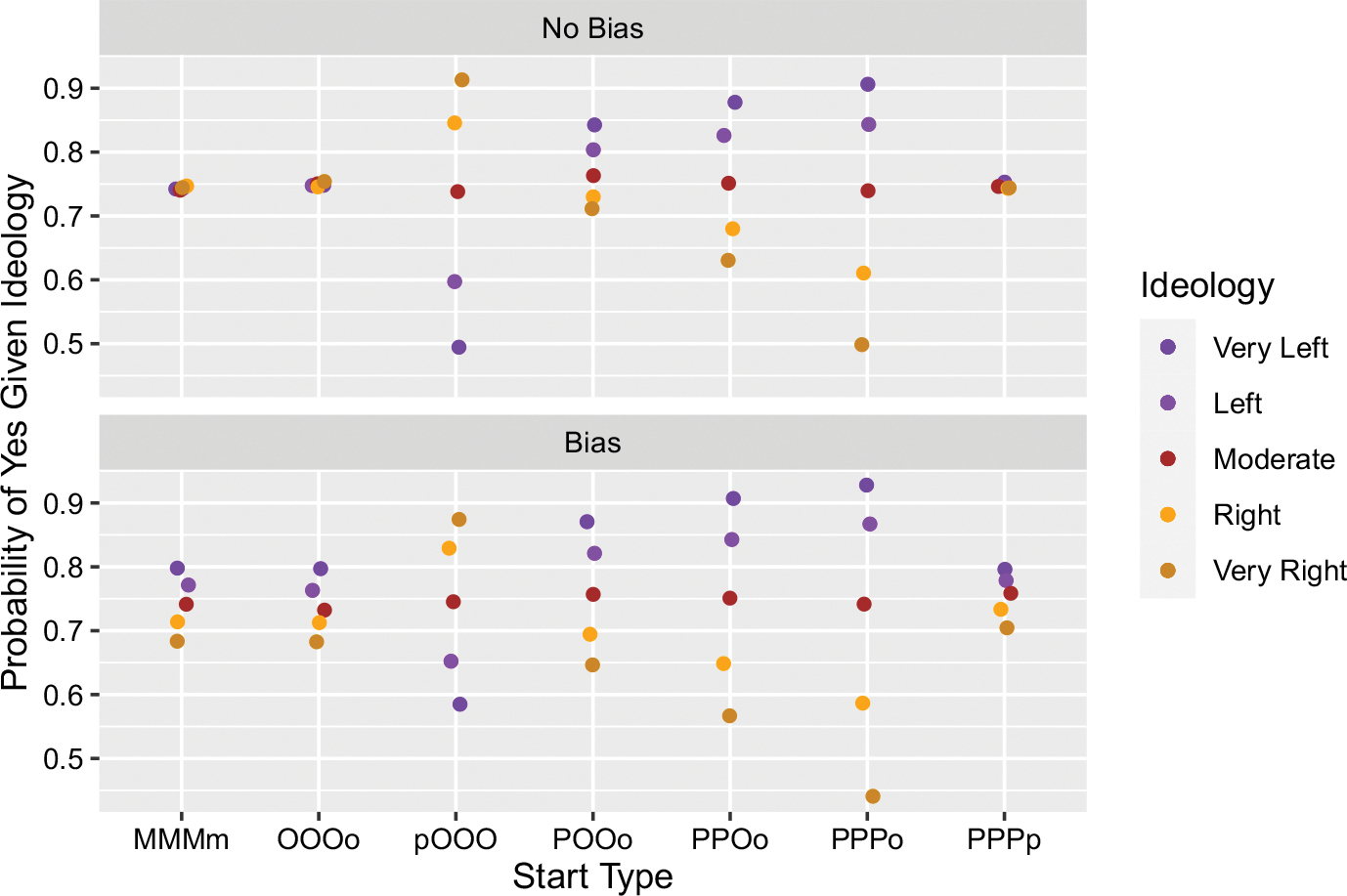
Results from simulations where three “Yes” beliefs and one “No” belief were initially distributed in different ways. (Notation as above in Fig. 2.)

Figure 3 shows seven different configurations of seeding three belief-yes and one belief-no.^11^

Similar to what we saw above, if the seeded beliefs all occur in the same group (as in MMMm, OOOo, and PPPp) then there is no polarization, and the resulting conditional probabilities of believing Yes reflect these starting conditions, i.e. 75% of each group, and hence 75% of the community, has belief-yes. If, however, we introduce motivated bias, then we see some polarization.

The starting conditions POOo, PPOo, and PPPo show, again, how polarization can emerge even in the absence of bias. Each of these conditions is a different way of distributing three token belief-yes when there is already an orange agent with a belief-no. We can see each of these distributions as increasingly favoring the spread of belief-yes among purple agents over orange ones, where POOo weakly favors belief-yes among purple, PPOo more so, and PPPo most strongly favors belief-yes among purples. We can see that the emerging polarization in each of these three conditions is also increasingly wider (which the introduction of motivated bias widens even further).

Perhaps the most interesting case is again where the initial seeding of beliefs is incongruent with agent ideologies: pOOO. We can see that polarization emerges absent motivated bias, and it is just as wide as the case of PPPo. The introduction of motivated bias, however, makes it so that ideologically incongruent beliefs are more difficult to spread in the respective groups. This leads to a throttling of the polarization dynamic that happens in the absence of motivated bias.

We now have two types of starting conditions where the ordering of the conditional probabilities is the opposite of the others, such that the orange are more likely to believe “Yes” and the purple less so (see ppOO and pOOO). This is as one would expect if initial starting situations play a prominent role in determining which beliefs get spread. If the orange start with “No” they’ll spread more “No” among each other, if they start with “Yes” they’ll spread more “Yes”. Mutatis mutandis for purple. This leads to the question: even if we have incongruent starts, i.e. the orange (purple) start with “Yes” (“No”), are there any levels of motivated bias that force beliefs to spread in such a way that they end up being largely congruent with ideology? That is, can motivated bias be high enough so that it not only throttles the polarization dynamic in one direction, but ultimately reverses it in the other?

#### 3.2.3 Incongruent starts and motivated bias

**Fig. 4:**
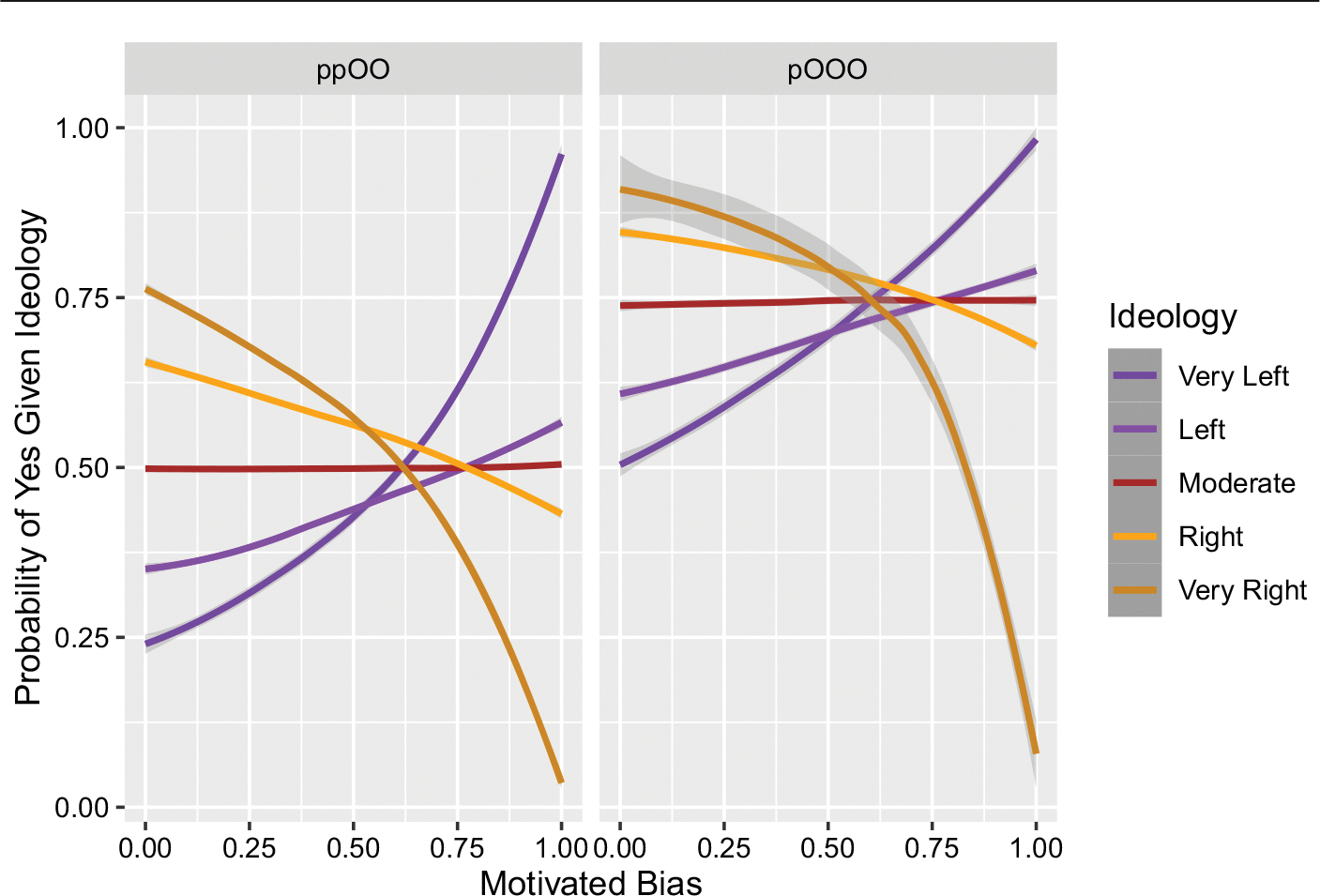
Incongruent starts. Some agents are given beliefs that are incongruent with their ideology and we then explore how much motivated bias is needed to result in belief spread that is largely congruent with ideology. For strong ideologies (Very Left and Very Right) this happens at about 62.5%, while for the weak ideologies (Left and Right) this happens at about 75% (reminder: that is the level that the strong ideologies reject, while weak ideologies are half that). Actual levels of motivated bias ranged from 0 to 1 in 0.1 increments. Each parameter setting was simulated 1,000 times

Figure 4 shows that the answer is yes: there are sufficiently high levels of motivated bias that can have incongruent starts but still lead to a spread of beliefs that are congruent with ideology. Depending on which level of ideology one looks at in the simulations, this reversal happens at levels of motivated bias of around 0.625–0.75. In the model, this means that the strong ideologies reject an invitation to form an incongruent belief about 62.5% of the time. The weak ideologies become largely congruent when the motivated bias for the strong ideologies is at 75% (which means the weak ideologies are rejecting about 37.5% of the time).

These simulations corroborate the idea that motivated bias, as a mechanism of exclusion, can be a powerful source of echo chambers, so much so that it can overpower what would otherwise be the spread of incongruent beliefs. How much motivated bias is required is easier to say in our simulations than in the real world: it is admittedly unclear how one would go about empirically quantifying a motivated bias of, say, 75%, not to mention issues about controlling variables in experimentation. Nevertheless, we can make at least some qualitative claims. When motivated bias is high, polarization happens so that beliefs and ideology are congruent. But when motivated bias is low, it is possible for polarization to happen for both congruent and incongruent beliefs. In the case where polarization occurs among incongruent beliefs, the explanation in our model is that this emerges from seeding beliefs that are initially “nano-polarized”.

## 4 Limitations and robustness

A tacit assumption about our model concerns the relationship between the speed at which things spread in the network and the rate at which information is available to the network as a whole. Specifically, our model does not consider the possibility that further information could be introduced that would change the minds of agents even after they have formulated their initial beliefs. Consequently, our agents do not end up changing their minds from “Yes” to “No” (or vice versa). In principle, however, our agents *could* change their minds if new information would supplant their belief through some kind of justificatory elevation that supports the contrary. There are many ways one could try to model this that we do not consider here.^12^ To that end, our results are limited to situations in which the rate at which news becomes available to a network is slower than the rate at which news is shared in the network.

Nevertheless, one may be concerned that our model misrepresents the relation between the speed at which information is shared and the speed at which agents update their beliefs. Perhaps, for example, when an agent receives news about some topic, they wait at least some short amount of time to see if others also have news to share, and only then does an agent form a belief and then avow that to their neighbors. In that kind of process, our agents *could* change their beliefs as news spreads through the network. Early on an agent might have a few neighbors reporting “Yes” (“No”) but then later more neighbors reporting “No” (“Yes”). If an agent updates their beliefs according to something like “adopt whatever the current majority of my neighbors are avowing”, then it’s possible that an agent does switch from “Yes” to “No” (or vice versa). In turn, they would also update their avowal, which could further cause neighboring agents to change their beliefs as majorities “flip”, and so on. Such a possibility is not unreasonable, even given the previous limitation about the rate of information introduced to the network. So we would like to know how robust our results are with respect to such a possibility—call it the *local majority updating* version of our model.^13^

**Fig. 5:**
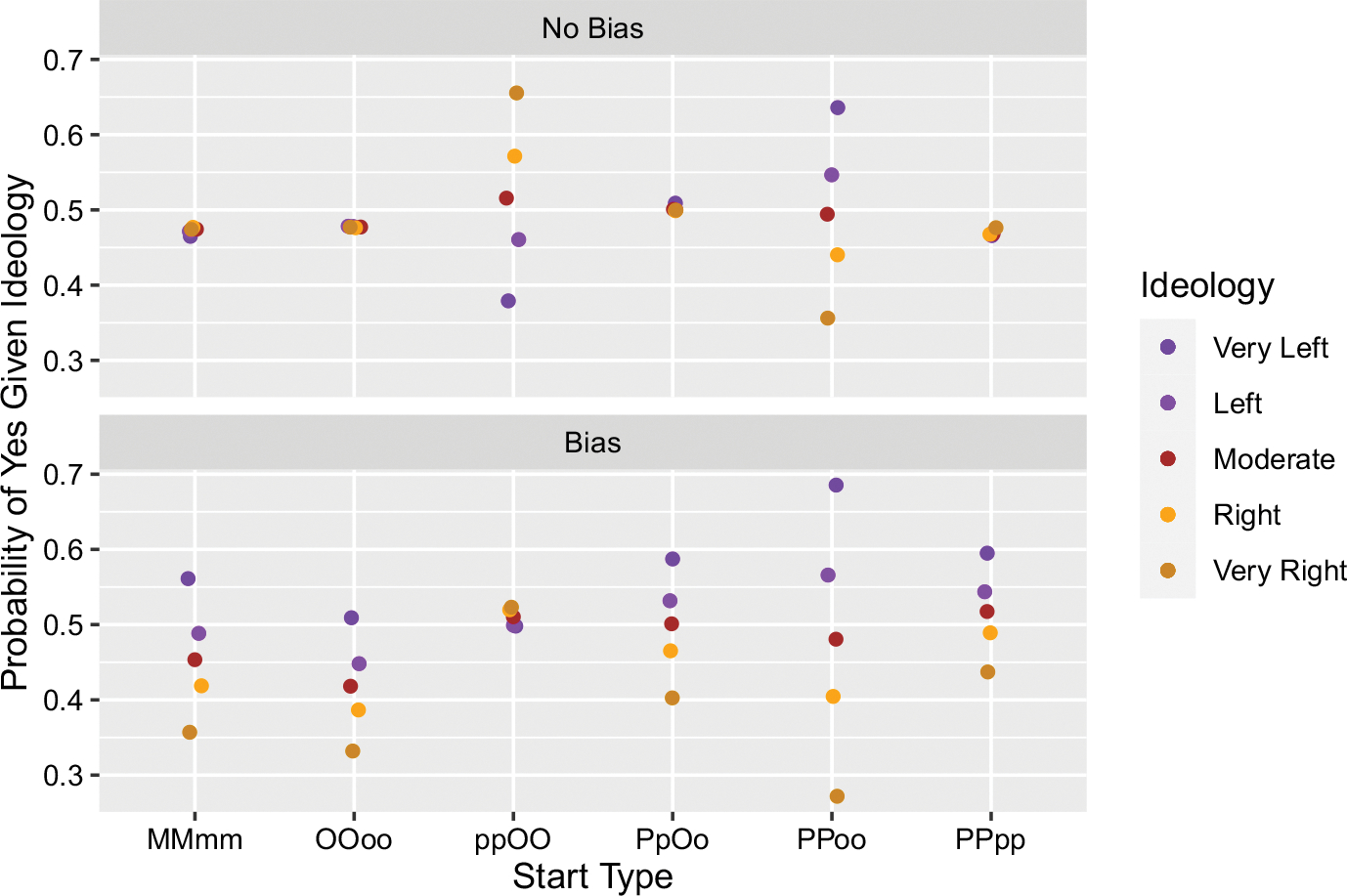
Results from our robustness check simulations where two “Yes” beliefs and two “No” beliefs were initially distributed in different ways. All distributions are nearly identical with the exception of ppOO with Bias, where here we no longer see polarization as before

Leaving further details and figures to Appendix A, our results are qualitiatively the same with two exceptions, and only slightly different quantitatively. With respect to quantity, recall that when there is no motivated bias we get issue polarization when the initial information is given disproportionately across two groups, e.g. two “Yes” to the orange side and two “No” to the purple. Exchanging our original belief spread process with the local majority one still produces polarization in these scenarios, though it is not quite as extreme (roughly 10% less in the most extreme cases—compare scenarios ppOO and PPoo across Figs. 2 and 5).

The first qualitative exception concerns the scenario where we have symmetric starts and the addition of motivated bias. Our original result saw issue polarization in this particular scenario, with a range from about 0.3 for the Very Left and 0.7 for the Very Right of saying “Yes” (see ppOO in the lower panel in Fig. 2). But in the local majority model we find the group tends towards a 0.5 probability of saying “Yes” given their ideology (see ppOO in the lower panel in Fig. 5). What explains this? Recall that adding motivated bias “pushes” beliefs in the direction of being congruent with ideology by limiting the adoption of incongruent ones. The effect is that polarization (1) emerges where there was none before, (2) increases polarization where there was some before, and (3) dampens polarization when it had emerged in spite of incongruency (as in the case of ppOO). When we add bias to the local majority version, there is a kind of suppression from those avowing a belief incongruent with a hearer’s ideology. This suppression makes it comparatively more difficult for incongruent beliefs to spread to such agents than to those where the beliefs would be congruent. Such “vote suppression” is not possible in the original version, and in the local majority version it only makes a difference in this one scenario where the “vote suppression” is all on one ideological side—in the case of PpOo, for example, the suppression happens equally on both sides and so balances out as far as the emergence of polarization goes.

**Fig. 6:**
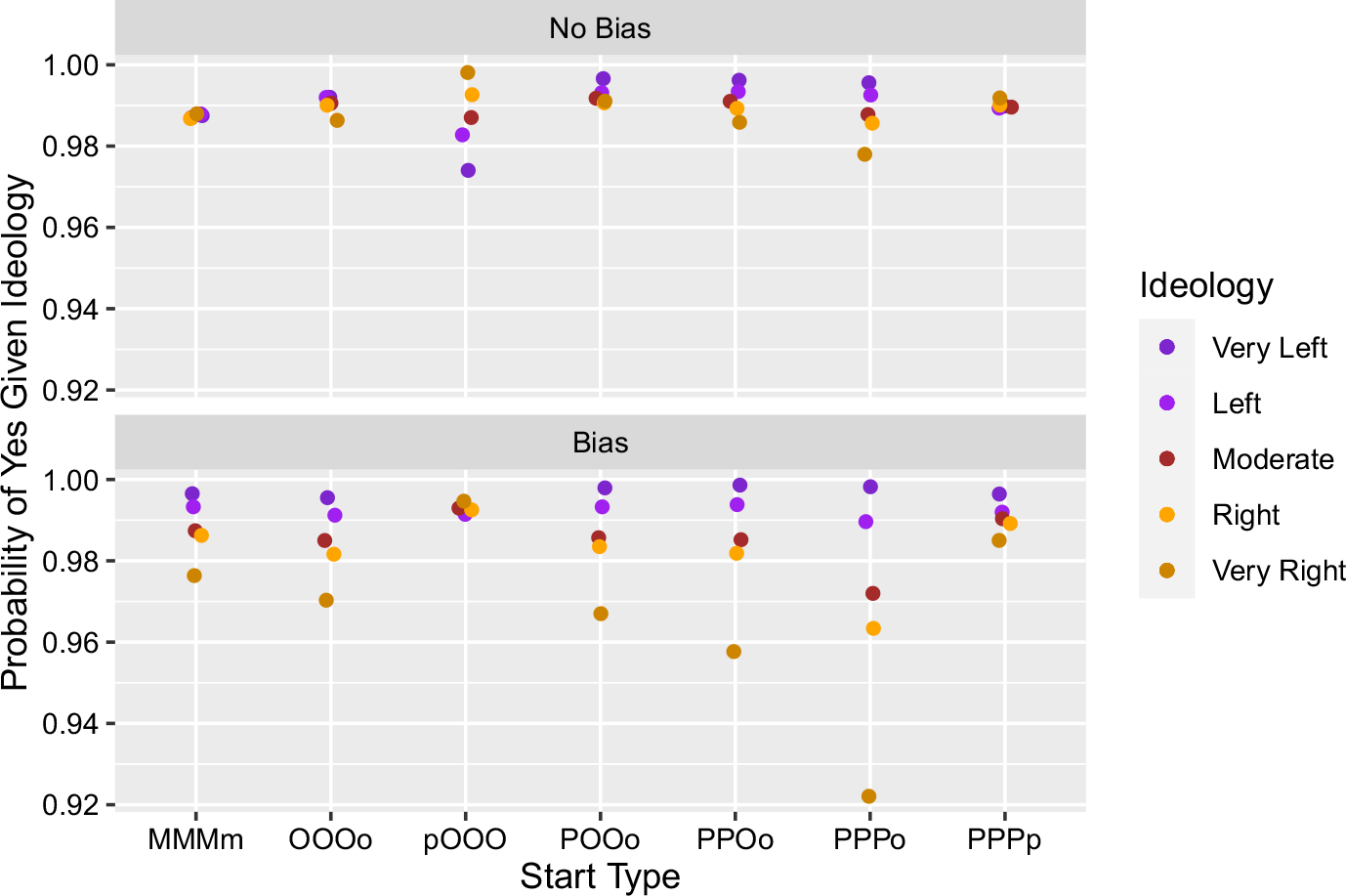
Results from our robustness check simulations where three “Yes” beliefs and one “No” belief were initially distributed in different ways. Notice in particular the y-axis: now agents are approaching consensus

This explanation similarly accounts for the second qualitative exception we see. In the original version we saw that asymmetric starts could still generate polarization (see Fig. 3). In the local majority version, however, we see near consensus across all the start types, with the lowest value of 0.92 of saying “Yes” (see Fig. 6). Again, switching to the local majority version means that “No” will tend to be in the local minority, and consequently, it will be both less likely to spread and provide “Yes” a significant advantage in a way that it did not have in the original model. The process very much resembles the Condorcet Theorem, but specific comparisons here would take us too far afield.

Relatedly, in our original model we asked whether incongruent starts could be overturned by motivated bias and we found the answer to be in the affirmative, so long as motivated bias was high enough. That is still true, with two interesting caveats. In the symmetric case the local majority version does not require as much motivated bias to reverse the incongruency—which is consistent with the “vote suppression” explanation above. In the asymmetric case we still get polarization for similarly high values of motivated bias, but when motivated bias is lower we no longer get “incongruent polarization” but rather the near-consensus just explained.

There are, of course, many other kinds of processes one could explore beyond the local majority updating version we implemented. The point here is to demonstrate that our findings are not dependent on the contingent fact that, in our original model, agents happen to not change their minds after forming their initial beliefs.

## 5 Implications and conclusion

Let us take a step back and take a broader look at things. Some explanations of persistent misperception of information and issue polarization appeal to social media environments, which lend themselves to group polarization (homophily) and echo chambers.^14^ The phrase ‘echo chamber’ has at least two inter-related meanings. One is a mechanism, the other a phenomenon. The mechanism is something like an amplification process in which a group of individuals increase their confidence in a view by sorting themselves in a way that leads to an echo effect in a chamber of discussion. As a phenomenon it is an in-group consensus of its own making, and when this happens for two subgroups of a population (‘group polarization’) the result is polarization on some issue, typically along lines that are congruent with ideologies. Variations of similar ideas include filter bubbles, information cocoons, epistemic bubbles, or personalized communication.

Analyses of these phenomena and the mechanisms undergirding them have demonstrated that there are interesting and subtle differences, both in understanding what the phenomena are and the mechanisms responsible for them. The subtle differences matter, both descriptively and prescriptively.

For example, C. Thi Nguyen argues that much of the literature conflates what Nguyen calls echo chambers and epistemic bubbles (Nguyen, 2020). While both are structures of exclusion, they differ in their origins and mechanisms for operation. Epistemic bubbles exclude voices from being heard by (accidental) omission through processes of social selection and community formation, whereas echo chambers exclude by actively discrediting and undermining ‘outside’ voices. The distinction is important for both descriptive and normative reasons. Descriptively, the two phenomena will have different behaviors: epistemic bubbles will burst with the introduction of contrary evidence, while that same evidence can intensify an echo chamber. Normatively, they call for different kinds of interventions.

Relatedly, according to Nguyen the mechanisms that undergird epistemic bubbles and echo chambers differ. Epistemic bubbles are not resilient against exposure to outside views because the mechanisms that generate them are not self-reinforcing towards the belief system. Echo chambers, on the other hand, are formed by a set of belief-supporting strategies that make it possible for the beliefs to survive multiple contacts with contrary views and evidence. Drawing on the work of Jamieson and Cappella (2008), Nyguyen’s analysis suggests that these belief-supporting strategies are such that exposure to contrary information can actually strengthen the belief system, as opposed to weaken it. If Nguyen is right, then the Millian imperative to improve society by facilitating dialogue across different views will work for epistemic bubbles, but could worsen the associated maladies in the case of echo chambers.

The idea that exposure to contrary information can actually reinforce someone’s view is sometimes known as *the backfire effect* (Nyhan & Reifler, 2010). While there was initially a great deal of excitement about this idea as an explanation for political misinformation and issue polarization, follow up studies have found the phenomenon to be quite nuanced. While the existence of the backfire effect is not rejected, it is not believed that backfiring occurs as frequently and as generally as was initially thought, and consequently, that its explanatory support of (political) misperception has been overstated (Nyhan, 2021).

That said, the Nguyen conception of echo chambers need not require that belief-supporting strategies *strengthen* a belief system in the face of contrary information; an echo chamber just needs *some* kind of mechanism that helps beliefs survive exposure to contrary information. To that end there are long-standing traditions in social psychology that study such mechanisms, such as biased assimilation (going back to at least Lord et al., 1979) or motivated reasoning (the 2006 Taber & Lodge, 2006 paper being the classic in the political context). In these traditions there is an invited presumption to see processes like biased assimilation as largely irrational. But more recent research has begun exploring mechanisms that are more nuanced.

For example, one mechanism that explains echo chambers and different forms of polarization is the preference for certainty, which has been offered by political psychologists in the related study of political homophily in social networks.^15^ A preference for certainty is a cluster of traits in which a person seeks certainty, stability and familiarity. While all humans have some preference for certainty, different levels of it have been found to be associated with differences in ideology. One (weaker) association is asymmetric: conservatives tend to have higher levels of a preference for certainty than liberals (Jost et al., 2003). Another (stronger) association is symmetric on the left-right ideology spectrum: ideological extremes tend to have higher levels of preference for certainty than moderates. In a recent study of political homophily on social networks (Twitter specifically), Boutyline and Willer found support for the claim that individuals that are ideologically more extreme will exhibit more political homophily than those at the center (Boutyline & Willer, 2017). Support was not found for the claim that the ideological right exhibited more homophily than the left. Other studies have found that while conservatives do have a higher preference for certainty in some domains, this preference does not carry over into differences in how they form epistemic networks in contrast to liberals (Guay & Johnston, 2020).

What is notable about a preference for certainty is that it carves out yet another phenomenon that does not fit neatly on Nguyen’s spectrum between epistemic bubbles and echo chambers, nor for that matter in the biased assimilation literature. The sensitivity that a bubble has to bursting is connected to the degree that the disagreement-reinforcement mechanism is absent. And the heart of this mechanism is the discrediting of individuals and institutions that offer information that is contrary to a person’s beliefs. However, unlike an epistemic bubble, a group of agents with a preference for certainty can maintain a belief system’s resilience against contact with contrary viewpoints, but also unlike an echo chamber (in Nguyen’s sense) or biased assimilation, this resilience need not come from the discrediting of ‘outside’ individuals or institutions. Instead, it could be that communities with high preference for certainty uphold prevalent beliefs on the basis of character traits that prioritize familiar attitudes and positions.

To our minds the preference for certainty sounds like an interesting avenue to explore. To do so requires some careful attention be paid towards the contributions made by the various traits that make up the preference for certainty, and to consider the extent to which these count as mechanisms of exclusion. It is easy, for example, to conflate various facets of what the preference for certainty is: some of the related traits of the preference for certainty are socially directed, others about assimilation of information. Such conflations are rightly criticized. Dandekar et al. (2013), for example, have argued that homophily alone may not generate polarization. Furthermore, Bramson et al. (2017) have outlined problems with using models of group polarization to understand observed polarization. Furthermore, the resulting conflation makes it difficult to identify interventions that are most appropriate (as argued by Nguyen).

In our analysis we have distinguished between two kinds of mechanisms: the cognitive and the social. We modeled the social side by a distribution of connections between agents of a community: while agents of all ideology types will have some connections with all ideology types, a slightly larger proportion of an agent’s connections will be with other agents that are similarly-minded.^16^ Rather than speculate what those proportions might be, we empirically validated our network to capture in a general way the structure of many epistemic communities. And importantly, agents in our model treat all connections on par in terms of who they are willing to listen to; and to this end agents are not participating in exclusionary practices. One might argue that anything except perfectly uniform connections across ideologies means there is some sense in which we have incorporated ideas about exclusion. Perhaps, but we worry that “exclusion” loses its explanatory power, for polarization does not always occur in our model (even though on average our epistemic communities have the same structure). Moreover, one cannot assess the claim that a mechanism of exclusion is driving the bubble or echo chamber without being sufficiently precise about the relevant thresholds that differentiate network structures. And even then, extant analyses are incomplete with respect to the possibility that polarization and echo chambers can be produced as an artifact of an ordering effect (more below).

On the cognitive side, we introduced the preference for belief, the idea that in the absence of defeaters, an agent will come to believe what they are invited to believe by someone in their epistemic community. This could be considered as a simplified version of the preference for certainty that focuses specifically on cognitive traits, but it also encompasses a variety of philosophical principles of belief updating, which lends at least some support in being considered reasonable. Importantly, a key feature of the preference for belief is that, again, it is not a mechanism of exclusion in the usual sense, such as discounting “untrusthworty” sources or views that are incongruent with one’s ideology. Rather, the preference for belief is, as are a host of related mechanisms, susceptible to an ordering effect that comes with removing the why-not element: first heard and believed, second heard, not believed. Perhaps that is enough to say that the preference for belief is a mechanism of exclusion by proxy, since after all, the acceptance of a belief automatically becomes a defeater for a set of other beliefs. Maybe so, but what matters to us is that this mechanism is not conflated with the kinds of exclusionary processes that are presumed in the sorts of analyses just discussed, such as biased assimilation or the backfire effect: the same set of interventions there will not have the same kinds of impacts on the mechanism that we have modeled. And moreover, it is less obvious that the preference for belief is epistemically unjustified, contrary to other cognitive biases. So our concern is that lumping the preference for belief in with other exclusionary processes invites a conflation we think should be avoided.

Bringing the social and cognitive mechanisms together, we have shown that the dynamic interaction between these two mechanisms can magnify particular micro-level belief inequalities into full blown polarization. This is not to say that standard mechanisms of exclusion do not play a role in explaining real-world echo chambers and polarization. In fact, inspired by mechanisms like biased assimilation and motivated reasoning, we have shown how we can incorporate a kind of exclusion mechanism in the form of motivated bias—the probability that an agent ignores an invitation to believe something that is incongruent with their ideology. And moreover, we have shown how at extremely high levels, motivated bias can not only throttle, but also reverse the direction of polarization given initial conditions to the contrary.

Our appeal to a dynamic interaction as an important explanatory source of issue polarization is consistent with some findings from the emerging study of *rational* polarization. For example, Kelly (2008) argues that differences in the histories of agents can have different causal effects on their evidence, which in turn can generate belief polarization, even if we otherwise suppose such agents are ideally rational. Another example is Singer et al. (2019), who argue that ‘coherence-mindedness’—prioritizing reasons for a view that is best supported by *all* the reasons an agent has access to—is epistemically rational given the limitations that humans have with respect to memory. They then use simulation studies to show how group polarization can emerge from coherence-mindedness. What these sorts of studies highlight is that polarization (both belief and group versions) can emerge due to a kind of path-dependence. We have similarly shown how a preference for belief (in ideological contexts) is subject to an ordering effect, which in turn can generate a kind of path-dependency as beliefs spread through a population.^17^

One goal of diagnosing the sources of issue polarization and echo chambers is to identify appropriate interventions (or if the sources are reasonable, then perhaps the lesson is for us to tolerate the outcomes). We believe our model has identified an important explanation that has been missed. Explanations that focus on mechanisms of exclusion will have associated interventions that suggest individuals be more “open-minded”, whether that be in a social sense (make sure you are not in a bubble) or a cognitive sense (make sure you don’t discount information or alternative sources simply because they are incongruent). But if some echo chambers and polarization emerge from the dynamic interaction of the preference for belief and structure of a community, both of which are not exclusionary in the sense above, then “be-more-open-minded” interventions will be unsuccessful. Unlike epistemic bubbles, exposure to contrary information will not reverse them, since in our model agents are exposed to contrary information. And unlike echo chambers the mechanism is not exclusion of the sources; in our network agents trust whoever they have connections with.

The kind of interventions that would be suggested by our model will be twofold—though significantly more work needs to be done before we would endorse these in practice. The first concerns the fact that the initial seeding of beliefs plays a prominent role in how beliefs spread and aggregate at the community level. Holding other things fixed, a difference in the initial beliefs is all it takes to have dramatically different outcomes. So great care needs to be taken in where information first enters a community. It is worth acknowledging that the communities we have modeled do not have “elite” agents, and this would be a natural next step to explore (in addition to loosening the grip on holding other things fixed).

The second concerns the fact that once some belief has spread, that belief becomes a defeater for the alternative belief. This suggests that in order to supplant the belief that has spread, it will require some kind of elevation of the second belief that goes beyond whatever warrant the first belief enjoys (this is consistent with Gilbert Harman’s discussion of belief perserverence in Harman, 1986, Chp. 4). In Section 4 we briefly considered how this might happen with a form of social deliberation, but we did not consider other forms of information gathering that might be ‘corrective’. Admittedly, however, it is not well understood how real cognitive systems handle corrective information—memory and timing complicate matters (Brashier et al., 2021), and we also know that the discrediting of evidence often has little effect (Ross & Anderson, 1982). That said, our insight is consistent with the idea that initial belief formation occurs with less cognitive effort than the process of using corrective information to prompt belief revision. Unfortunately, this means that the task of correcting misinformation is a rather difficult task, but extremely important if it is necessary for maintaining the foundation of a representative democracy (Jerit & Zhao, 2020).

In short, even without mechanisms of exclusion as standardly understood, echo chambers and issue polarization can emerge from a dynamic interaction between a preference for belief and an epistemic network. While mechanisms of exclusion may exacerbate polarization, they may not be the ultimate root of it. Hence, the recommended interventions suggested by extant literature on exclusion mechanisms is expected to be limited—even if we succeeded at addressing them, issue polarization can still quite easily occur. Issue polarization may reflect a failure to nip in the bud the early more innocent looking seedlings of polarization. Unfortunately, this means that the problem of issue polarization may be even more intractable than the current literature suggests; to boot, if the sources of issue polarization really are innocent and reasonable, interventions may not be warranted at all.

## A Robustness checks

We implemented the idea that agents “adopt whatever the current majority of my neighbors are avowing” as follows. At time step *t*, all agents with a “Yes” or “No” belief avow their respective belief to their neighbors. At the next time step *t* + 1, all agents (irrespective of their belief status) collect the avowels received at the previous step and adopt whichever is the majority (if there is one). Thus an agent that may have just avowed “Yes” at time *t* may come to adopt “No” at time *t* +1, and if so, will avow “No” at the next iteration of the cycle at *t* + 2. This process repeats until the beliefs of the community reach a steady state.^18^

We reproduced each of the simulation scenarios we presented in the main text, but using our local majority updating model instead of the original version. The figures in this section contain the respective plots.

There are all sorts of ways we could have modeled a social process. The primary goal of these supplemental simulations was to examine whether our results hung on the particular implementation by which agents update their belief by ‘consulting’ (so to speak) one agent at a time, or whether multiple agents could be consulted and then aggregated in some way. More specifically, we wanted to address a potential concern that our results are dependent on the contingent fact that our agents don’t happen to change their beliefs in our original version. What we see is that the social process we selected, local majority updating, leaves most of our initial results intact, but in some cases also introduces its own effects, particularly when there is an uneven distribution at the outset. And this is to our point, namely, that it is quite possible that issue polarization (and perhaps consensus in some scenarios) reflects the initial conditions of how information is brought into a network.

## Figures and Tables

**Fig. 7:**
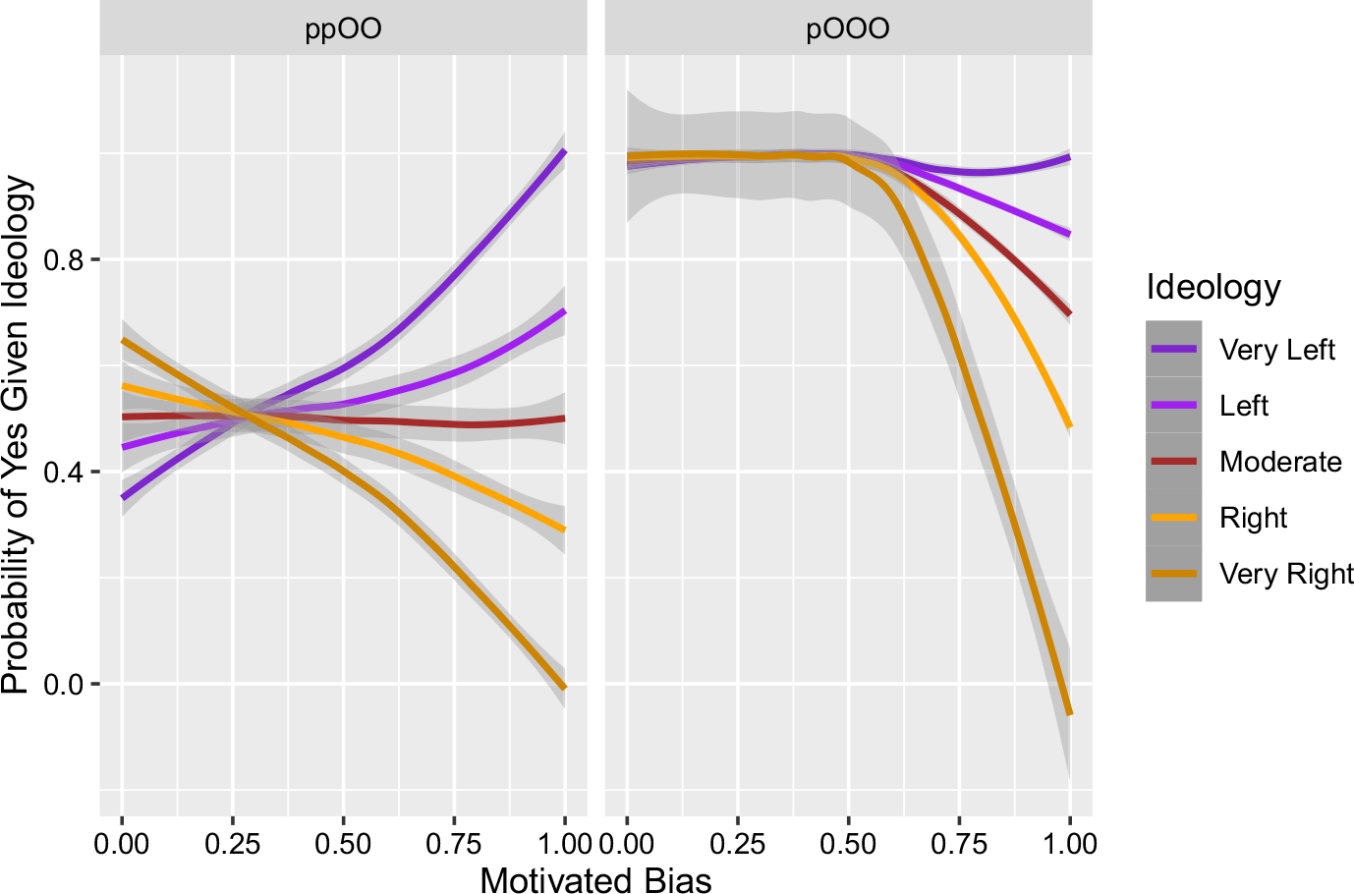
Incongruent starts. Some agents are given beliefs that are incongruent with their ideology and we then explore how much motivated bias is needed to result in belief spread that is largely congruent with ideology—here using our local majority updating version

**Table 1:** Characteristics of the ideology spectrum in our model given a network of 200 agents

No.	Ideology	Visual	Mean Frequency	Mean Bubble

1	Very Left	Deep Purple	25	0.75
2	Left	Purple	35	0.63
3	Moderate	Brown	70	0.53
4	Right	Orange	35	0.63
5	Very Right	Deep Orange	25	0.75

## Footnotes

1 Our example is by no means limited to presidential elections. The kinds of effects we are pointing to have been documented in other related respects: Kahan et al. (2017), Kahneman and Tversky (2013).

2 See Chapter 4 specifically.

3 See Chapter 2 of Orléan’s *Empire of Value* Orléan (2014).

4 See for example Clark and Chase (1972), Gilbert (1991), Gough (1965), Harris et al. (2008), Lewandowsky et al. (2012), and Millikan (2004).

5 Gilbert Harman’s discussion of the Principle of Positive Undermining in the context of belief perseverance is a nice example of how this could go (see Harman, 1986, Chp. 4).

6 Specifically, undirected links—the number of directed links would be twice that.

7 Simulations of our model were done using Netlogo 6.2.0 and can be found here: https://modelingcommons.org/browse/one_model/7060model_tabs_browse_info. Some robustness analysis was done using a different version of our model (discussed further below) and can be found here: https://modelingcommons.org/browse/one_model/7061model_tabs_browse_info. Analysis of simulation data was done using R version 1.4.1106 and the script can be found here: https://github.com/bertybaums/preference-for-belief.

8 Our epistemic communities are ideologically symmetrical and the yes/no beliefs are exclusive and exhaustive by the end of a simulation. It is potentially interesting to explore communities that are not ideologically symmetrical, in which case one would need to track both belief-yes and belief-no.

9 One might argue that “exclusion” comes in degrees and that the level of exclusion is proportional to our bubble-coefficient. So in a sense, we have incorporated ideas about exclusion. That is fine but does not detract from the point that analyses are incomplete with respect to the possibility that polarization and echo chambers can be produced as an artifact of an ordering effect. We discuss this point in more detail in Section 5.

10 The kind with one belief-yes and three belief-no is equivalent under the appropriate symmetrical transformations and so we omit those results.

11 Again, not shown are the start situations with three “No” beliefs and one “Yes”. These are symmetrically equivalent to the three “Yes” situations with the ideologies reversed—compare start situations pOOO and PPPo.

12 One complementary example is the exploration of group deliberation through ‘town meetings’, as illustrated by Pulick et al. (2016).

13 We thank an anonymous reviewer’s skepticism about the robustness and generalizability of our results for inspiring this version of our model.

14 Sometimes ‘issue polarization’ is also referred to as ‘belief polarization’. Both of these are about how beliefs form across a population, which is not to be confused with ‘group polarization’, which is about how social connections form between agents. There are other differences that don’t concern us much here, but see Bramson et al., (2017) for further distinctions.

15 See Gaffney et al. (2014) and Sherman et al. (2009) as examples of using the avoidance of uncertainty in explanations of polarization.

16 We are by no means the first to have explored the modeling of preferential associations between likemined agents and its connection to opinion polarization. A classic model here is the Hegselmann and Krause model Hegselmann et al. (2002). We see such work as complementary to ours, though at a higher level of abstraction.

17 Whether our model counts as an example of rational polarization is, to our minds, too early to say. The field is still in its infancy and it’s not clear to us yet what the relevant criteria are for counting as rational polarization. While we think, for example, that the preference for belief has support for being reasonable, we are neutral with respect to the other aspects of our model, such as the small amount of preferential associations between like-minded agents, and to what degree beliefs situated in (or sensitive to) ideological contexts are subject to rational scrutiny.

18 In very rare cases a steady state is not reached due to the configuration of the network and a particular pattern of beliefs. As an illustrative example, suppose you have a square with corners A, B, C, and D, such that A is connected to B and C but not D, and so on. At *t* let A and D have “No” beliefs and let B and C have “Yes” beliefs. Then at *t* +2 A and D will have adopted “Yes” beliefs while B and C will have adopted “No” beliefs. At *t* +4 the distribution of beliefs would be back to where they were at *t*, and the oscillation would continue indefinitely. However, such cases are extremely rare because network configurations are extremely unlikely to have the needed symmetries at larger scales, not to mention the very particular distributions of beliefs to get such oscillations. The reader is invited to work through a few simple examples to see for themselves.
